# Clinical Outcomes of Daytime Versus Nighttime Laparoscopic Appendectomy in Children

**DOI:** 10.3390/children10040750

**Published:** 2023-04-20

**Authors:** Zenon Pogorelić, Ivana Janković Marendić, Tin Čohadžić, Miro Jukić

**Affiliations:** 1 Department of Pediatric Surgery, University Hospital of Split, 21000 Split, Croatia; 2 Department of Surgery, School of Medicine, University of Split, 21000 Split, Croatia

**Keywords:** acute appendicitis, children, laparoscopic appendectomy, daytime surgery, nighttime surgery, shifts, outcomes of treatment, complications

## Abstract

*Aim of the study*: To evaluate the clinical outcomes and complication rate of laparoscopic appendectomy in children operated on during the daytime versus nighttime. *Methods*: A total of 303 children who underwent laparoscopic appendectomy for acute appendicitis between 1 January 2020 and 31 December 2022 were enrolled in this retrospective study. The patients were divided into two study groups. The first group consisted of the patients who underwent laparoscopic appendectomy during the day shift from 07:00–21:00 (*n* = 171), while the patients in the second group underwent laparoscopic appendectomy during the night shift from 21:00–07:00 (*n* = 132). The groups were compared for baseline clinical and laboratory data, treatment outcomes, and complications. The Mann–Whitney U test was used to compare continuous variables, while the Chi-square test was used to compare categorical variables. A two-sided Fisher’s exact test was used when the frequency of events in a certain cell was low. All *p* values less than 0.05 were considered significant. *Results*: The proportion of complicated appendicitis was almost the same in both patient groups (*n* = 63, 36.8% vs. *n* = 49, 37.1%, *p* = 0.960). Out of the total number of patients presenting during the daytime and nighttime, 11 (6.4%) and 10 (7.6%) developed a postoperative complication, respectively (*p* = 0.697). Additionally, rates of readmission (*n* = 5 (2.9%) vs. *n* = 2 (1.5%); *p* = 0.703), redo-surgery (*n* = 3 (1.7%) vs. *n* = 0; *p* = 0.260), conversion to open surgery (*n* = 0 vs. *n* = 1 (0.8%); *p* = 0.435) and length of hospital stay (*n* = 3 (IQR 1, 5) vs. *n* = 3 (IQR 2, 5); *p* = 0.368) did not differ significantly between daytime and nighttime appendectomies. The duration of the surgery was significantly shorter in patients presenting during the day than in those presenting at night (26 min (IQR 22, 40) vs. 37 min (31, 46); *p* < 0.001). *Conclusions*: Different shift times did not affect the treatment outcomes or complication rates for children receiving laparoscopic appendectomy.

## 1. Introduction

Acute appendicitis is caused by inflammation of the appendix and is one of the most common surgical emergencies in adults and children. Compared with adults, the pediatric population is at higher risk of complicated acute appendicitis, a reality which prolongs hospital stay [1,2]. The annual incidence of acute appendicitis is 19–28 per 10,000 for children under the age of 14, with an overall lifetime risk of 9% for males and 7% for females [3,4]. In the pediatric population, the incidence of acute appendicitis is highest between the ages of 12 and 17 years, with a peak incidence rate seen between the ages of 11 and 12 years [3,4]. The rates of perforation are significantly higher in younger children compared to older children and adolescents [1,3,4]. The lifetime probability of an appendectomy is 23% for females and 12% for males [2].

Acute appendicitis is the most common reason for abdominal surgery in children and can present in various stages as simple or complicated acute appendicitis [2]. Typical symptoms include anorexia, fever, vomiting, tenderness, and guarding in the right iliac fossa, with alternating pain beginning in the periumbilical region. In younger children, the onset of symptoms is usually atypical [5,6]. Various etiologies of appendicitis lead to luminal obstruction by an appendicolith, caused by increased mucus production and bacterial growth. Eventually, this condition leads to venous congestion, appendiceal wall tension, and necrosis, progressing to purulent inflammation and perforation. Subsequently, generalized peritonitis or appendiceal mass/abscess occurs [6]. Possible triggers for the mechanisms of acute appendicitis include the obstruction of the lumen by an appendicolith, tumor, lymphoid hyperplasia, foreign body obstruction, or viral infection leading to secondary bacterial infection [6,7].

The clinical presentation of acute appendicitis is usually typical in the majority of cases and highly noticeable during physical examination [8]. Other diagnostic procedures include history, laboratory tests, ultrasound, or even computed tomography scans. There are clinical scoring systems in use in the diagnosis of acute appendicitis, such as the appendicitis inflammatory response (AIR) score and the Alvarado score [8,9]. In addition to standard biomarkers, several new biomarkers for acute appendicitis, such as hyponatremia, hyperfibrinogenemia, hyperbilirubinemia, pentraxin-3 (PTX-3), neutrophil gelatinase-associated lipocalin (NGAL), or interleukin-6 (IL-6), have recently been investigated. These biomarkers showed good predictive values for the detection of acute appendicitis, as well as for distinguishing between complicated and simple acute appendicitis [10,11,12,13,14,15,16]. A more recent study showed that non-invasive markers from saliva, such as leucine-rich α-2-glycoprotein 1 (LRG1), may be promising biomarkers for the detection of acute appendicitis in children [17]. In addition, LRG1 proved to be an excellent serum biomarker with very high sensitivity and specificity in the detection of acute appendicitis in children [18].

A positive family history of acute appendicitis carries a 3-fold risk of developing the disease [1]. Appendectomy is considered an effective and safe treatment for acute appendicitis. Although antibiotic therapy is an effective and safe treatment for simple acute appendicitis, laparoscopic appendectomy is still considered the golden standard in the treatment of acute appendicitis [7]. According to the Dutch guidelines, surgery should be performed within the first 8 h when acute appendicitis is suspected [19]. 

Appendectomy can be performed using the open or laparoscopic approach [20,21]. The advantages of laparoscopy over open surgery are shorter hospital stays and less postoperative pain, but recovery from acute appendicitis depends mainly on whether the appendix was perforated or not during the procedure [1,5]. Postoperative complications, such as abscess formation or ileus are more common in patients with complicated appendicitis [1]. The possibility of postoperative wound infections is much lower with laparoscopic appendectomy [22]. After a laparoscopic appendectomy, return to the usual diet is faster and patients have good bowel function. Finally, laparoscopy is a good diagnostic method in cases of negative appendectomy [20].

A recent meta-analysis clearly demonstrated that delayed laparoscopic appendectomy (up to 24 h) does not increase the likelihood of developing complications in simple appendicitis [23]. In addition, previously published results suggest that surgeons may defer appendectomy in patients with suspected simple appendicitis occurring at night [23,24]. Another recent study has shown that short deferral of appendectomy does not increase the risk of appendiceal perforation, but may be associated with a slightly increased risk of surgical site infections [25,26]. In contrast to patients with simple appendicitis, delaying surgery for more than eight hours in patients with complicated acute appendicitis is associated with a higher risk of postoperative complications [27].

Laparoscopic appendectomy is performed as an emergency procedure in the majority of cases. The work of an on-call physician in late-night hours results in sleep deprivation, fatigue, lack of energy, and poor concentration. Patients with more severe clinical symptoms usually arrive at hospital at night and may therefore be sicker and have more complex pathologies. Theoretically, both reasons may affect postoperative outcomes for patients [3,28].

This paper aims to evaluate the clinical outcomes and complication rates of laparoscopic appendectomy in children operated on during the day or at night.

## 2. Materials and Methods

### 2.1. Patients

**Figure 1:**
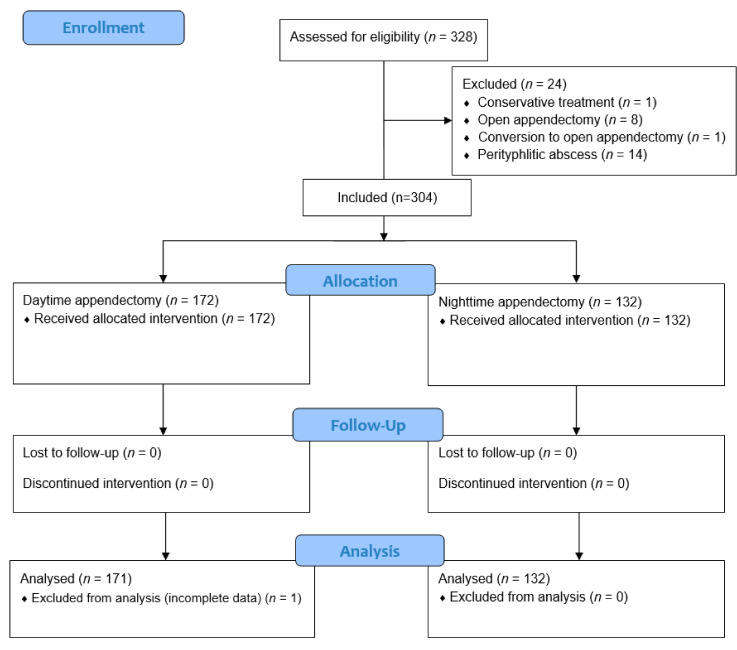
A flowchart diagram of the study.

All pediatric patients who underwent laparoscopic appendectomy for acute appendicitis were included in the retrospective study. The study was conducted in the Department of Pediatric Surgery, University Hospital of Split, between 1 January 2020 and 31 December 2022. The inclusion criteria were all pediatric patients (under 18 years of age) who underwent laparoscopic appendectomy for suspected acute appendicitis at our institution. The exclusion criteria were patients over 18 years of age, conservative treatment or/and primary open appendectomy, and incomplete data in case records. A total of 328 patients were assessed for eligibility. Finally, after the exclusion of the patients who did not meet the inclusion criteria, 303 patients were included for analysis (Figure 1).

### 2.2. Outcomes of the Study

The primary data obtained from the study was the rate of postoperative complications between the two study groups (daytime vs. nighttime). Secondary outcome measures were duration of surgery, readmission rate (ReAd) [29], unplanned return to the operating room (uROR) [30], conversion rate to open surgery, and duration of hospital stay. Surgical complications were classified according to the Clavien–Dindo scale [31].

### 2.3. General Policy of Treatment of Acute Appendicitis and Indications for Emergency Surgery

Our pediatric surgery department has no restrictions concerning the indication for nighttime emergency surgery. Our policy is to operate on children at the time of diagnosis in order to avoid pain and stress (which is different from our policy toward adult surgery). Additionally, during the study period, all cases of acute appendicitis were managed by a laparoscopic approach. At our department, laparoscopic appendectomy is the method of choice, even in cases of complicated appendicitis. The open approach is used only in exceptional circumstances. All cases of acute appendicitis in our institution are subjected to surgery. Only older children and adolescents with periappendicular abscess formation may be treated by a conservative approach and delayed appendectomy, usually 2–3 months after diagnosis.

### 2.4. Study Design

Patients were retrospectively divided into two study groups. The first group consisted of the patients who underwent laparoscopic appendectomy during the day shift from 07:00–21:00 (*n* = 171), while the patients in the second group underwent laparoscopic appendectomy during the night shift from 21:00–07:00 (*n* = 132). The groups were compared in terms of baseline patient demographics (age, sex, weight, and height), clinical characteristics (duration of symptoms, body temperature, vomiting, right lower quadrant pain, rebound tenderness, and AIR score), laboratory data (leukocyte count, C-reactive protein, and neutrophil granulocyte count), pathohistological analysis data from the appendectomy specimens removed, treatment outcomes, and complications. ReAd and uROR rates were also compared between the study groups. The indication for surgery and the diagnosis of acute appendicitis were made by the operating pediatric surgeon based on a combination of clinical examination, laboratory data, AIR score, and findings on abdominal ultrasound.

### 2.5. Surgical Technique

All patients included in the study underwent a standard 3-port laparoscopic approach. A Veress needle was used to establish a CO_2_ pneumoperitoneum, ranging from 6 to 12 mmHg, depending on the characteristics of the patient. First, a 5 mm trocar was placed in the supraumbilical region. After the introduction of the endoscope (Olympus, Tokyo, Japan) an initial inspection of the abdominal cavity is performed. The second trocar (5 mm) was placed in the right upper abdominal quadrant, and a third trocar (10 mm) is placed in the left lower abdominal quadrant. After visualization of the appendix and relief of inflammatory adhesions, the appendix was dissected using a harmonic scalpel (Lotus™; BOWA-electronic GmbH, Gomaringen, Germany) [32]. The appendiceal base was secured using a polymer clip (Ligating Clips XL; Grena, Brentford, London, UK) [33] or by repeated applications of a harmonic scalpel in a stepwise manner in order to obliterate the lumen of the appendix, as described in our previous study [34]. After the abdominal cavity was rinsed with saline, the appendix was retrieved through the 10 mm trocar using an endoscopic bag (Ecosac EMP 70; Espiner Medical Ltd.; Measham, UK). At the end of the procedure, the stump was checked for leakage before retracting all trocars and performing skin closure.

### 2.6. Postoperative Protocol and Follow-Up

After the surgical procedure, intravenous fluid (Ringer’s lactate or normal saline) was administered until oral intake began. Oral nutrition was started a few hours after surgery, depending on the surgeon’s decision. For analgesia, paracetamol (Paracetamol Kabi, Fresenius Kabi, Zagreb, Croatia) was used at a dose of 10–15 mg/kg or ibuprofen (Brufen, Mylan, Zagreb, Croatia) at a dose of 10 mg/kg. Antibiotic therapy was used in all patients with complicated acute appendicitis. In most cases, this was a combination of gentamicin (Gentamicin, Belupo, Koprivnica, Croatia) at a dose of 3–5 mg/kg, administered once daily, and metronidazole (Metronidazole B. Braun, B. Braun Adria, Zagreb, Croatia) at a dose of 7.5 mg/kg, administered three times daily. In cases where this antibiotic combination was not effective, the antibiotic therapeutic method was changed according to the antibiogram. In the majority of cases, patients with simple appendicitis did not receive antibiotic therapy. Afebrile patients without significant pain, vomiting, or fever and with complete tolerance of oral meals were discharged from the hospital within 24 h. These measures were in accordance with our standard protocol, which was published earlier [2]. Children were followed up in our outpatient clinic 7 and 30 days after discharge.

### 2.7. Ethical Aspects

The study was conducted according to the specifications of the Declaration of Helsinki of the World Medical Association and approved by the ethics committee of our hospital (protocol code 500-03/22-01/188; date of approval: 28 November 2022).

### 2.8. Statistical Analysis

Statistical analyses were performed using SPSS 28.0 (IBM Corp., Armonk, NY, USA) and Microsoft Excel for Windows version 11.0 (Microsoft Corporation, Redmond, WA, USA) software. The D’Agostino–Pearson test was used to determine the normality of the data distribution. The median and IQR or mean ± standard deviation (SD) were used to describe the distribution of quantitative data, while categorical data were described using absolute numbers and percentages. Depending on the normality of data distribution, the independent *t* test or its nonparametric alternative, the Mann–Whitney U test, were used to compare continuous variables, while the Chi-square test was used to compare categorical variables. A two-sided Fisher’s exact test was used when the frequency of events in a certain cell was low. All *p* values were two-tailed, and *p* values less than 0.05 were considered to be significant.

## 3. Results

### 3.1. Baseline Characteristics, Demographic and Clinical Data of the Study

**Figure 2:**
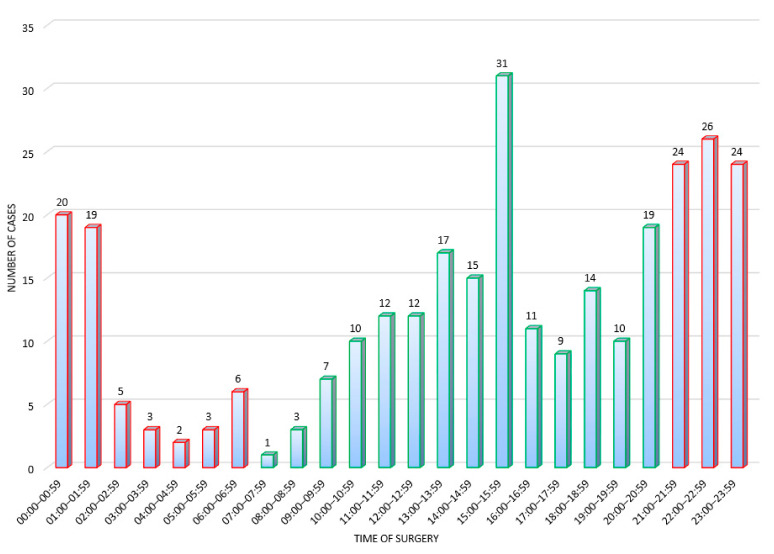
The hour proportion of patients with acute appendicitis in regard to the time of surgery (green bars—daytime; red bars—nighttime).

A total of 303 pediatric patients (196 (64.7%) male) who underwent laparoscopic appendectomy during the study period and met the inclusion criteria were enrolled in the study. The median age of all children included in the study was 11 (interquartile range, IQR 8, 15) years. The appendix was removed during the day in 171 (56.4%) of the patients and during the night in 132 (43.6%). The largest number of patients (*n* = 31) underwent surgery between 15:00 and 15:59 (Figure 2).

The majority of patients (97.3%) had no concomitant diseases, while epilepsy and diabetes mellitus were the most common concomitant diseases among patients with concomitant diseases. The demographic characteristics and clinical and laboratory data of the patients are shown in Table 1. No statistically significant differences were found between the groups in terms of demographic data: age (*p* = 0.631), gender (*p* = 0.287), weight (*p* = 0.865), and height (*p* = 0.703). Additionally, no statistically significant differences were found between the groups in terms of clinical and laboratory data: duration of symptoms (*p* = 0.667), body temperature (*p* = 0.853), vomiting (*p* = 0.778), right lower abdominal quadrant pain (*p* > 0.999), rebound tenderness (*p* = 0.520), AIR score (*p* = 0.833), leukocytes (*p* = 0.700), C-reactive protein (*p* = 0.412), and neutrophil count (*p* = 0.556).

### 3.2. Pathohistological Analysis

The intraoperative findings were positive for acute appendicitis in 274 (90.5%) patients, of whom 66 (24.1%) had complicated appendicitis. The pathohistological analysis of the removed specimens in the acute appendicitis group showed that the majority of patients (*n* = 125; 41.3%) had phlegmonous appendicitis, whereas gangrenous appendicitis was found in 109 (36%) of patients. Catarrhal appendicitis was found in 30 (9.9%) patients, whereas appendicitis was caused by carcinoid in 3 (1%) patients. A comparison of the pathohistological analysis between the study groups is shown in Table 2.

### 3.3. Comparison of Study Outcomes

No statistical difference was found between the study groups regarding the primary outcome of the study and complication rate (Table 3). The total number of complications in patients who underwent appendectomy during the daytime was 11 (6.4%), while the number of complications in patients who underwent laparoscopic appendectomy during the nighttime was 9 (6.8%) (*p* = 0.679). The most common complication in both groups was an abscess (*n* = 7; 4.1% vs. *n* = 4; 3%). Duration of postoperative fewer within 72 h of surgery was similar in both study groups (*n* = 11 (6.4%) vs. *n* = 10 (7.6%); *p* = 0.697). Rates of ReAd (*n* = 5 (2.9%) vs. *n* = 2 (1.5%); *p* = 0.703), uROR (*n* = 3 (1.7%) vs. *n* = 0; *p* = 0.260), conversion to open surgery (*n* = 0 vs. *n* = 1 (0.8%); *p* = 0.435) and length of hospital stay (*n* = 3 (IQR 1, 5) vs. *n* = 3 (IQR 2, 5); *p* = 0.368) did not differ significantly between the groups. The duration of the surgery was significantly shorter in patients presenting during the day (26 min (IQR 22, 40) vs. 37 min (31, 46); *p* < 0.001).

Only one immediate intraoperative complication was registered—a bladder injury during the first trocar insertion in a child with a full bladder. It was a small perforation which was resolved during the same surgery with one laparoscopic stitch and urinary catheter placement, and the patient recovered without further complications. The classification of complications according to the Clavien–Dindo classification is presented in Table 4.

## 4. Discussion

The results of this retrospective study showed no difference in clinical outcome and complication rate in children in whom laparoscopic appendectomy was performed during the day or at night. Because appendectomy is one of the most commonly performed surgeries in the pediatric population, numerous studies have been conducted in order to ensure the safety of each step of the procedure. However, few studies have considered the impact of the time of day at which the surgery is performed on postoperative complication rates, readmission, and redo-surgery rates, the need for conversion to open surgery, duration of surgery, length of hospital stay, and overall patient care. Therefore, the purpose of this study was to determine whether different shift times affect the aforementioned outcomes.

In terms of complication rates, this study confirmed the safety of nighttime appendectomy, with no significant differences between study groups. Similar to previous publications, the most common complication in both study groups was abscess formation [3,24,35]. Other postoperative complications such as ileus, wound infections, and bleeding at the trocar insertion site were less common. In this study, we did not find any intraoperative complications in daytime surgeries; however, a single intraoperative complication in nighttime surgeries was reported as a bladder injury. The study also provided insight into how different shift times might affect the number of readmissions and unplanned readmissions to the operating room after laparoscopic appendectomy. The potential impact of the time of day on these two parameters was important because they have been described as indicators of good quality of care in several studies, including those conducted at our institution [29,30]. These studies also defined appendectomy as a major cause of readmission and unplanned reoperation in pediatric surgery. Nevertheless, we were unable to identify nighttime appendectomy as a contributing factor to readmission or reoperation. However, complicated appendicitis, open surgical technique, and diabetes mellitus remain the most important factors associated with 30-day readmission after appendectomy [29,30,36,37]. Nowadays, laparoscopic appendectomy has become the main treatment method for acute appendicitis and is often considered to be a relatively simple and quick operation. In some cases of complicated appendicitis, retrocecal location of the appendix, or severe bleeding when the surgeon has limited ability to see the anatomy of the appendix and surrounding structures, conversion to laparotomy is indicated [38]. It has been documented that demographic factors such as age > 13 years and male gender, as well as obesity and appendicitis with subsequent perforation or diffuse peritonitis, contribute to a higher frequency of conversion to laparotomy, which is then associated with higher postoperative morbidity [39,40].

In this study, no cases were reported of conversion to open surgery during the day, and there was only one conversion during the night. Given the low incidence of conversion to laparotomy, it is safe to say that our results are consistent with recent publications describing decreasing rates of conversion to open surgery [39]. Numerous studies have examined in detail the advantages and disadvantages of open and laparoscopic appendectomy, with conflicting results regarding the duration of these procedures. Some studies suggest that open surgery takes less time, while others consider laparoscopic surgery to be faster [20,41]. In this study, laparoscopic surgery performed during the day was found to take significantly less time than that performed at night. Nevertheless, nighttime laparoscopic appendectomy was not associated with increased operative time because it was performed within the standard time frame suitable for this procedure [42]. Contrary to some reports, there was no association between the time of day and length of hospital stay in our patients [1,16]. The average hospital stay was three days in both groups, confirming the results of previous studies in our department [20].

Although appendectomy remains the gold standard in the treatment of acute simple appendicitis, several studies advocate the safety of conservative treatment. This is associated with lower costs and a lower complication rate than surgical treatment, but may come with a longer hospital stay and a higher rate of appendicitis recurrence [43,44,45]. Another controversy in the management of acute appendicitis is whether appendectomy can be safely delayed until the next day. In patients without preoperative signs of complicated appendicitis, studies have confirmed that it is safe to delay appendectomy for up to 24 h, whereas patients with complicated appendicitis should be taken to the operating room immediately regardless of the time of day [23,25]. For this reason, studies on how different shift times affect clinical outcomes are essential. A study of 1643 pediatric patients by San Basilio et al. showed that nighttime appendectomy was as efficient as daytime surgery in terms of complication rate, length of hospital stay, and readmission rate. Laparotomy was the preferred surgical approach during the night, with a significantly shorter operative time. Compared with our results, the authors reported higher complication and readmission rates, with a one-day shorter hospital stay for all groups, finding the most common complication to be an abscess, which is consistent with our results [3]. Mönttinen et al. included both children and adults in their retrospective study and found that nighttime appendectomy had no differences in morbidity, mortality, duration of surgery, and extent of intraoperative bleeding compared with appendectomy performed during the daytime. Reported complications were consistent with our findings, describing a shorter length of hospital stay for patients who underwent surgery at night [24].

On the other hand, another study by Shah et al. reported a longer hospital stay for patients undergoing a nighttime appendectomy [1]. Half of the patients waited more than 8 h from admission to surgery, and the waiting time from admission to the operating room was significantly longer for patients who underwent surgery during the night shift. Most patients underwent surgery between 7 pm and 1 am, and there was no difference in the clinical outcomes between patients operated on during the day and those operated on at night [1]. A study of adult patients by Patel et al. showed that complication rates in patients operated on at night were comparable to those of patients operated on during the day. Delaying surgery by up to 24 h was also shown to be safe, with no increased risk of complications noticed [46]. In contrast to previous studies, Bom et al. found that nighttime appendectomy was associated with a higher risk of complications, especially in the subgroup of complicated appendicitis, with most complications being due to wound infections. Children were excluded from this study, and most operations were performed laparoscopically. The number of reoperations was significantly higher in patients those who were hospitalized at night, whereas there was no difference in readmission between daytime and nighttime operations, similar to the results of our study.

In contrast to the results of Patel et al., waiting time from admission to the operating room of more than 8 h on the day shift was found to be associated with a significantly higher risk of postoperative complications. Such an association was not found in those who underwent surgery during the night. Consistent with the aforementioned results, the average hospital duration of stay for both groups was two days, and no cases of mortality were reported in patients operated on at night [28]. Tago et al. conducted a study of patients diagnosed with acute appendicitis at night (5:30 pm to 8:30 am) and compared the demographic, operative, and postoperative characteristics of two groups, those who underwent an appendectomy at night and those who waited until daytime to undergo surgery. Their study showed that it was safer to delay surgery until daytime, as these patients had a significantly lower risk of surgical site infections than those who underwent nighttime appendectomy within the same hospital stay [47].

Several studies in different surgical emergency departments reached controversial conclusions. Some suggested that nighttime surgery was a risk factor for intraoperative complications with little effect on morbidity and mortality [48], while others associated it with a higher risk of morbidity and death [49,50]. A study in thoracic surgery found that lung resections performed using a video-assisted thoracoscopic surgery approach (VATS) had a significantly higher incidence of intraoperative complications when performed during the night shift [51]. In a study by Buget et al. of children operated on for supracondylar humerus fracture, better outcomes were found with daytime operations, with shorter operative time and lower morbidity than with nighttime operations [52]. Studies in various fields of abdominal surgery again supported the safety of daytime surgery [53,54,55,56]. It was found that cholecystectomy performed at night was as successful as that performed during the day, with no difference observable in complication rates or mortality. Additionally, it was observed that the length of hospital stay was similar in patients in both groups, but that patients operated on at night were more likely to undergo laparotomy [54,55,56].

Possible unfavorable outcomes of nighttime surgeries could have been due to increased sleepiness and fatigue of on-call physicians, but also because patients with more serious pathologies were not able to wait until daytime to come to the hospital. Tomasko et al. showed that, despite the increased sleepiness and workload of on-call surgeons working night shifts, this did not affect task completion and learning of new skills [57]. Others noted that tasks with lower cognitive demands were easily accomplished by sleep-deprived surgeons, whereas the performance of tasks with higher cognitive demands in such circumstances was more problematic as cognitive functions continuously declined with less sleep [58,59]. Reports on the effects of fatigue on surgical outcomes conflicted widely and left ample room for further investigation [30,60,61,62]. Several studies showed no significant association between the surgeons operating the night before and not operating the previous night for treatment outcomes [63,64].

The limitations of this study should be considered. This is a single-center study with a retrospective analysis. Surgeons’ experience and the effects of fatigue and sleep deprivation on their skills were not assessed in this study, meaning that further research is needed to determine their influence on postoperative outcomes. However, the data displayed were quite comprehensive, and postoperative outcomes were carefully recorded for all our patients. Additionally, several children with comorbidities were included which could potentially influence the results of the study. To avoid bias all of types, the records were double-checked and these children with comorbidities were found not to have any complications; this is the reason why we decided to include them in the study. Because the number of patients included in this study was not particularly large, a multicenter study design based on a larger patient population is needed to shed more light on these outcomes.

We would like to reiterate that the time of day should not cause a delay in the immediate care of patients requiring emergency surgery. Nevertheless, postponing surgery to the daytime in nonemergency cases may improve patient comfort, lead to faster recovery, and shorten the length of hospital stay. Given the conflicting results of various publications, further research is needed to gain more insight into these issues.

## 5. Conclusions

This study has confirmed the safety of nighttime appendectomy compared with daytime appendectomy, with comparable complication rates and clinical outcomes being obtained for all pediatric patients receiving laparoscopic appendectomies. The quality of care remains intact regardless of the different shift times.

## Figures and Tables

**Table 1:** Demographic characteristics, clinical and laboratory data of patients.

	Group I (*n* = 171) Daytime Appendectomy	Group II (*n* = 132) Nighttime Appendectomy	*p*
Demographic Characteristics of Patients; Median (IQR) or *n* (%)			
Age (years)	11 (9, 14)	11 (8, 15)	0.631 *
Gender			
Male Female	115 (67.3) 56 (32.7)	81 (61.4) 51 (38.6)	0.287 ^†^
Weight (kg)	45 (34, 60)	44 (35, 65.5)	0.865 *
Height (cm)	159 (141, 174)	156.5 (143, 174)	0.703 *
Clinical data of patients; median (IQR); mean ± SD or *n* (%)			
Duration of symptoms (h)	22 (20, 44)	24 (20, 48)	0.667 *
Body temperature (°C)	37.1 ± 0.7	37.2 ± 0.8	0.853 ^§^
Vomiting	97 (56.7)	77 (58.3)	0.778 ^†^
Pain in RLQ	171 (100)	132 (100)	>0.999 ^‡^
Rebound tenderness	141 (82.5)	105 (79.5)	0.520 ^†^
AIR score; median (IQR)	7 (4, 8)	7 (4, 8)	0.833 *
Laboratory data of patients; median (IQR) or mean ± SD			
Leukocytes (×10^9^/L)	14.4 ± 4.7	14.7 ± 5.5	0.700 ^§^
C-reactive protein (mg/L)	17.9 (6, 44.9)	17.1 (5.6, 43.3)	0.412 *
Neutrophil granulocytes (%)	80.7 ± 10.3	80.0 ± 9.8	0.556 ^§^

* Mann–Whitney U-test; ^†^ chi-square test; ^‡^ Fisher’s exact test; ^§^ independent *t* test; BMI—body mass index; IQR—interquartile range; RLQ—right lower quadrant; AIR—appendicitis inflammatory response; SD—standard deviation.

**Table 2:** Pathohistological analysis of removed specimens.

Variables *n (%)*	Group I (*n* = 167) Daytime Appendectomy	Group II (*n* = 129) Nighttime Appendectomy	*p*
Catarrhal appendicitis	17 (10.2)	13 (10.1)	0.976 *
Phlegmonous appendicitis	74 (44.3)	51 (39.5)	0.409 *
Gangrenous appendicitis	62 (37.1)	47 (36.4)	0.902 *
Neuroendocrine tumor	1 (0.6)	2 (1.6)	0.582 ^†^
No appendicitis	13 (7.8)	16 (12.4)	0.185 *

Missing data: group I (*n* = 4), group II (*n* = 3); * Chi-square test; ^†^ Fisher’s exact test.

**Table 3:** Treatment outcomes between the groups.

Variables Median (IQR) or *n* (%)	Group I (*n* = 171)	Group II (n = 132)	*p*
	Daytime Appendectomy	Nighttime Appendectomy	
Postoperative fever < 72 h	11 (6.4)	10 (7.6)	0.697 *
Complications (total)	11 (6.4)	9 (6.8)	0.893 *
Postoperative ileus	2 (1.2)	1 (0.8)	>0.999 ^†^
Abscess	7 (4.1)	4 (3)	0.761 ^†^
Wound infection	1 (0.6)	2 (1.5)	0.582 ^†^
Bladder injury	0 (0)	1 (0.8)	0.435 ^†^
Bleeding—trocar insertion site	1 (0.6)	1 (0.8)	>0.999 ^†^
Duration of surgery (min)	26 (22, 40)	37 (31, 46)	<0.001 ^‡^
Re-admission	5 (2.9)	2 (1.5)	0.703 ^†^
Redo-surgery	3 (1.7)	0 (0)	0.260 ^†^
Conversion to laparotomy	0 (0)	1 (0.8)	0.435 ^†^
Duration of hospital stay (days)	3 (1, 5)	3 (2, 5)	0.368 ^‡^

* Chi-square test; ^†^ Fisher’s exact test; ^‡^ Mann–Whitney U-test; IQR—interquartile range.

**Table 4:** The Clavien–Dindo classification of postoperative complications.

Grade	Group I (n = 171)	Group II (n = 132)	*p* *
	Daytime Appendectomy	Nighttime Appendectomy	
I	2 (1.2)	1 (0.8)	>0.999
II	5 (2.9)	3 (2.3)	>0.999
III a	1 (0.6)	2 (1.5)	0.581
III b	3 (1.8)	3 (2.3)	>0.999
IV a	0	0	-
IV b	0	0	-
V	0	0	-

* Fisher’s exact test.

## Data Availability

The data presented in this study is available upon request of the respective author. Due to the protection of personal data, the data is not publicly available.
